# Early-Phase Quadriceps Activation After Knee Surgery: A Narrative Review of Current Rehabilitation Interventions and Identification of an Unmet Clinical Need

**DOI:** 10.3390/jcm15134903

**Published:** 2026-06-24

**Authors:** Abdulmajeed Alfayyadh

**Affiliations:** Department of Physical Therapy and Health Rehabilitation, College of Applied Medical Sciences, Jouf University, Sakakah 72388, Saudi Arabia; abalfayyadh@ju.edu.sa

**Keywords:** quadriceps rehabilitation, arthrogenic muscle inhibition, early postoperative rehabilitation, knee surgery, neuromuscular inhibition, home exercise, rehabilitation technology

## Abstract

Arthrogenic muscle inhibition (AMI), neurophysiological suppression of voluntary quadriceps activation triggered by joint effusion and inflammation, is consistently initiated within hours of any form of knee surgery. If not actively counteracted during the first two postoperative weeks, AMI may drive a cascade of neuromuscular, morphological, and biomechanical deficits that can persist for years, substantially increasing the risk of post-traumatic osteoarthritis, reinjury, and long-term functional disability. Emerging evidence indicates that preoperative patient-related factors, including baseline quadriceps strength, age, body mass index, and physical fitness, further modulate the rehabilitation response and should be considered in planning early postoperative protocols. This narrative review, which was not designed as a systematic review or meta-analysis and therefore does not include formal quality assessment or pooled statistical analysis, evaluates evidence for seven early-phase (0–2 weeks postoperative) knee muscle activation interventions: neuromuscular electrical stimulation (NMES), isometric quadriceps exercise, blood flow restriction (BFR) training, electromyographic (EMG) biofeedback, open and closed kinetic chain (OKC/CKC) exercise, cryotherapy, and continuous passive motion (CPM). Findings are synthesized against six clinically relevant dimensions, safety in the 0–2 week window, home-based usability, capacity to overcome AMI, requirement for volitional effort, objective monitoring capability, and progressive resistance, to characterize a consistent pattern: no single existing modality simultaneously meets all combined requirements for home deployment, volitional engagement, objective monitoring, and progressive resistance from postoperative day one. This collective unmet need provides direction for future device development and clinical research.

## 1. Introduction

Knee surgery, encompassing anterior cruciate ligament (ACL) reconstruction, total knee arthroplasty (TKA), meniscal repair, meniscectomy, and cartilage procedures, is among the most frequently performed orthopaedic interventions globally, with more than 250,000 ACL reconstructions and over 700,000 TKA procedures performed annually in the United States alone [1]. Irrespective of the specific procedure, all forms of knee surgery trigger a common neurophysiological cascade within hours of the operation. Joint effusion and inflammatory mediators sensitize group III and IV articular afferents, initiating reflex inhibition of the quadriceps femoris through spinal and supraspinal mechanisms collectively termed arthrogenic muscle inhibition (AMI) [2]. As little as 20–30 mL of intra-articular fluid is sufficient to reduce voluntary quadriceps activation by up to 50%, driven by presynaptic inhibition and altered corticospinal excitability [3]. Without targeted intervention, AMI may persist for months to years, initiating a cascade of muscular atrophy, gait compensation, and articular loading abnormalities that substantially elevate the lifetime risk of post-traumatic osteoarthritis and reinjury [4,5].

The clinical mandate for early quadriceps activation is well established. The 2023 Aspetar Clinical Practice Guideline for ACL reconstruction rehabilitation, developed using Grading of Recommendations, Assessment, Development and Evaluations (GRADE) methodology, explicitly identifies volitional quadriceps activation as the primary rehabilitation objective in weeks 0–2, ahead of all other goals [6]. The OPTIKNEE consensus similarly identifies quadriceps strength symmetry as the most critical early rehabilitation target [7]. Longitudinal data confirm why: quadriceps strength at 12 weeks postoperatively explains approximately 47% of the variance in strength at return-to-sport testing [8], and persistent weakness at one year is associated with the development of radiographic knee osteoarthritis [4,9].

Despite this evidence base, a clinical gap persists between what early rehabilitation requires and what currently available tools can deliver, particularly outside supervised clinical settings. The purpose of this narrative review, which uses informal, thematic synthesis methods and should not be interpreted as a systematic review or meta-analysis, is to evaluate existing evidence for early-phase knee muscle activation interventions across all forms of knee surgery, identify the specific clinical limitations of each modality, and characterize the collective unmet clinical need that these limitations define. The review also considers preoperative patient-related factors that modulate rehabilitation response and discusses directions for future research addressing the identified gap.

## 2. Search Strategy

Electronic databases including PubMed, MEDLINE, the Cochrane Library, Web of Science, and EMBASE were searched for peer-reviewed literature published primarily between 2015 and 2025. Search terms were combined using Boolean operators and included: quadriceps rehabilitation, arthrogenic muscle inhibition, early postoperative rehabilitation, knee surgery, neuromuscular electrical stimulation, blood flow restriction, biofeedback, isometric exercise, open kinetic chain, closed kinetic chain, cryotherapy, continuous passive motion, total knee arthroplasty, ACL reconstruction, meniscal repair, preoperative factors, and rehabilitation predictors. Priority was given to randomized controlled trials (RCTs), systematic reviews, meta-analyses, and clinical practice guidelines. Foundational mechanistic studies published before 2015 were included where no more recent equivalent existed. The 0–2 week postoperative window was the primary temporal focus, with later timepoints referenced where they inform the mechanistic basis of early rehabilitation decisions. No formal protocol was registered for this narrative review, and no quality appraisal tool was systematically applied; this constitutes a methodological limitation and means findings should be interpreted as a synthesis of the available literature rather than definitive evidence ranking.

## 3. Preoperative Patient-Related Factors Influencing Early Rehabilitation Response

Before examining specific postoperative interventions, it is clinically important to acknowledge that the trajectory of early rehabilitation after knee surgery is substantially shaped by patient-level factors present before surgery. These variables modulate both the severity of early AMI and the patient’s capacity to respond to rehabilitation, and should be considered when designing and interpreting early-phase intervention protocols [10,11].

Preoperative quadriceps strength is among the strongest modifiable predictors of postoperative outcomes. Across studies of ACL reconstruction, deficits in preoperative quadriceps strength have been independently associated with inferior knee function at two years [11], lower probability of return to sport [12], and slower quadriceps strength recovery at one year postoperatively [13]. A prospective cohort study of 646 patients undergoing ACLR demonstrated that the combination of age greater than 22.5 years, female sex, body mass index (BMI) greater than 24.3 kg/m^2^, lower pre-injury sport level, and preoperative quadriceps limb symmetry index below 63.3% collectively and independently predicted failure to achieve adequate quadriceps strength recovery at one year (area under the curve = 0.73) [13]. These findings suggest that preoperative screening should encompass not only quadriceps strength but also anthropometric and activity-level variables to identify patients at highest risk of early AMI severity and poor rehabilitation response.

Age and BMI exert additional independent effects on postoperative rehabilitation outcomes. In TKA populations, higher BMI and older age have been consistently identified as predictors of slower functional recovery, with one prospective study of 362 TKA patients demonstrating that BMI, age-adjusted comorbidity index, and sleep quality were significant predictors of poor recovery at one year (area under the curve = 0.802) [14]. Physical fitness and prior sports participation similarly modulate early rehabilitation capacity. Patients with higher pre-injury activity levels demonstrate better baseline neuromuscular function, which facilitates both volitional quadriceps activation and adherence to rehabilitation protocols in the immediate postoperative period [15].

The clinical implications are twofold. First, patients with identified preoperative risk factors, older age, elevated BMI, low physical fitness, lower baseline quadriceps strength, and reduced sport participation, may require more intensive or more frequent early-phase rehabilitation support, including adjunct modalities such as NMES to overcome severe AMI. Second, preoperative exercise programs aimed at improving quadriceps strength before surgery (prehabilitation) have shown potential for improving postoperative strength recovery, though the persistence of these benefits beyond the immediate postoperative period remains unclear and warrants further investigation [16]. Acknowledging these patient-related factors contextualizes the evidence for each intervention reviewed in the subsequent sections and highlights the need for individualized, rather than uniform, early-phase rehabilitation protocols.

## 4. The Critical Importance of the 0–2 Week Postoperative Period

The first two postoperative weeks represent a period of significant neurological vulnerability and may offer important leverage for long-term outcome modification. Quadriceps strength at 12 weeks post-ACL reconstruction explains approximately 47% of the variance in strength at return-to-sport testing [8]. A systematic review and meta-analysis with longitudinal modelling confirmed that knee extensor strength remains meaningfully reduced, greater than 10%, at one year in a majority of patients who fail to achieve early strength targets [17].

The biological basis for this temporal sensitivity is AMI consolidation. Joint effusion activates presynaptic inhibitory pathways within hours of surgery and, if unchecked, may produce cellular-level changes in quadriceps architecture including fiber-type shifts, satellite cell depletion, and mitochondrial dysfunction that become progressively more difficult to reverse over time [18,19]. A prospective multicenter study documented that AMI Grade ≥ 1 occurred in 36% (95% CI 30–42%) of patients at two weeks post-arthroplasty, with female gender (OR = 2.81), high pain scores (OR = 13.57), and positional habits such as keeping the knee bent independently associated with its occurrence [20]. Concurrent cortical reorganization alters the sensorimotor representation of the quadriceps: transcranial magnetic stimulation studies document increased intracortical inhibition and decreased corticospinal excitability that can persist beyond two years if not targeted in the early postoperative period [21].

The TKA population illustrates the magnitude of this challenge: one month after surgery, quadriceps strength is approximately 40% of preoperative values, and voluntary activation is approximately 82% of preoperative levels despite physiotherapy beginning on postoperative day one [22]. Crucially, NMES initiated within 48 h of TKA, but not NMES initiated at four weeks, significantly attenuated this early strength decline [22], suggesting that the physiological mechanisms associated with AMI may be more amenable to intervention in the immediate postoperative window. These principles appear to apply across knee surgery types, providing a general rationale for early activation across procedures, though it is acknowledged that direct comparative evidence across all surgery types remains limited.

## 5. Arthrogenic Muscle Inhibition: Mechanisms and Treatment Principles

AMI is defined as the inability to fully activate a muscle voluntarily following injury or surgery to the adjacent joint, in the absence of direct muscle damage [2]. At the spinal level, abnormal discharge from joint mechanoreceptors and nociceptors, sensitized by effusion, inflammation, and tissue disruption, activates inhibitory interneurons that reduce efferent motor drive to the quadriceps via presynaptic mechanisms [3]. Supraspinal contributions include altered cortical sensorimotor mapping, increased intracortical inhibition, and decreased corticospinal excitability documented by transcranial magnetic stimulation studies within days of ACL injury [21]. Lepley and colleagues further identified a biopsychosocial dimension, showing that pain catastrophizing and kinesiophobia may amplify neural inhibition beyond the purely reflexive mechanism [21]. Importantly, AMI varies in severity between individuals and is influenced by the patient-related factors outlined in Section 3, including age, sex, and baseline functional status [20].

Sonnery-Cottet and colleagues proposed a clinical classification of AMI severity (Grades 0–3) based on vastus medialis oblique contraction quality and extension deficit severity, providing a practical framework for guiding treatment intensity [2]. Therapeutically, AMI may be partially overcome through NMES (which bypasses inhibited voluntary pathways by directly depolarizing motor axons), cryotherapy (which reduces effusion-mediated afferent drive), joint aspiration where clinically appropriate, and targeted volitional activation exercises reinforced with real-time feedback [2,3,23]. A central principle emerging from contemporary AMI research is that rehabilitation tools must address the neurological component of quadriceps dysfunction, with devices demanding active, controlled, measurable contractions against progressive resistance potentially facilitating motor engram retraining while normalizing afferent–efferent communication [24]. Whether any specific device achieves this more effectively than standard protocols remains to be established through prospective clinical trials.

## 6. Current Early-Phase Rehabilitation Interventions

### 6.1. Neuromuscular Electrical Stimulation

NMES is the most extensively studied early-phase modality and carries the strongest evidence base for quadriceps recovery following knee surgery. By directly depolarizing motor axons, NMES produces quadriceps contractions independent of central motor drive, uniquely bypassing the inhibited voluntary activation pathway [25,26]. A landmark RCT by Stevens-Lapsley and colleagues (n = 66) demonstrated that NMES initiated 48 h after TKA attenuated quadriceps strength loss and significantly improved functional performance, including stair climbing, timed up-and-go (TUG), and six-minute walk tests, at 3.5 weeks, with effects persisting to one year [22]. Quantitative synthesis confirms the magnitude of these effects: a 2025 systematic review and meta-analysis of 11 RCTs (n = 402) found NMES combined with standard rehabilitation significantly increased quadriceps muscle strength at both short-term (≤6 weeks: SMD 0.53, 95% CI 0.27–0.79) and long-term (>6 weeks: SMD 0.59, 95% CI 0.18–0.99) follow-up after ACL surgery (*p* < 0.001), with subgroup analysis showing that initiation within one week produced greater benefit than later initiation [25,26]. In TKA patients, a meta-analysis of 14 RCTs documented effect sizes of SMD 0.81 (95% CI 0.51–1.11) within one month, declining to SMD 0.46 (95% CI 0.18–0.74) at 12–13 months [27], indicating greatest responsiveness in the immediate postoperative period. A more recent single-center RCT (n = 37) confirmed that NMES commenced on postoperative day two produced significantly better isometric strength at four and eight weeks compared to standard rehabilitation alone [28].

Despite this evidence base, NMES carries substantive practical limitations in the 0–2 week postoperative window. Clinical-grade NMES devices require professional administration, electrode placement expertise, and individualized intensity calibration. While home NMES units exist, they require supervised calibration to achieve therapeutic intensities, and evidence suggests that sub-maximal stimulation reduces efficacy. Critically, NMES is fundamentally passive: the patient generates no volitional effort, limiting its contribution to motor re-learning and restoration of patient agency. NMES also provides no objective performance feedback regarding repetition quality or completion across sessions. Inconsistency across NMES protocols, varying frequency, intensity, duration, and timing, contributes to heterogeneity across studies and limits the strength of clinical recommendations [25,27]. Nevertheless, NMES represents the most evidence-supported single modality for the 0–2 week window, and its passive activation mechanism appears to offer complementary rather than redundant value alongside volitional exercise protocols.

### 6.2. Isometric Quadriceps Exercises

Isometric quadriceps exercises, principally the quadriceps set and straight leg raise (SLR), form the foundation of 0–2 week rehabilitation protocols across all contemporary clinical guidelines [6,7]. The foundational RCT evidence was provided by Shaw and colleagues (n = 103 ACLR patients), who demonstrated that performing SLRs and isometric contractions throughout the first two postoperative weeks significantly improved knee range of motion (*p* = 0.01–0.04) and was associated with a significantly lower incidence of abnormal laxity at six months, without compromising graft integrity [29]. At six months, the exercise group reported significantly better Cincinnati Symptom and sport-related outcome scores [29]. A 2024 evidence-based framework confirmed favorable EMG activation patterns, showing substantial VMO, VL, and rectus femoris activity with minimal ACL strain during quad set exercises [30].

Isometric exercises are universally accessible, inexpensive, and safe across all knee surgery types. However, a critical and consistent limitation is the complete absence of objective performance monitoring. No standardized tool currently documents the number of correctly completed repetitions, the level of effort applied, or the consistency of exertion across sessions. Research consistently demonstrates that home exercise adherence after knee surgery is highly variable: one cross-sectional survey of 204 TKA patients found self-reported completion rates as low as 30%, with day-to-day fluctuations in pain, mood, and self-efficacy identified as primary drivers rather than stable patient characteristics [31]. This monitoring gap is clinically significant because sub-therapeutic engagement with home exercise programs is essentially undetectable by the treating clinician. Compared with NMES, which produces direct muscle activation regardless of patient effort, isometric exercise relies entirely on patient-generated effort that is currently unverifiable outside supervised settings, a key distinction that has received insufficient attention in the early rehabilitation literature.

### 6.3. Blood Flow Restriction Training

BFR training employs partial venous occlusion via a pneumatic cuff to produce muscle hypertrophy and strength gains at low loads (20–40% of one-repetition maximum), generating results comparable to high-load resistance training at 70–80% of one-repetition maximum in non-surgical populations [32]. In knee rehabilitation, BFR has generated considerable interest as a mid-phase tool: a 2025 meta-analysis of middle-aged and elderly knee patients found that preoperative BFR training significantly enhanced postoperative muscle strength (SMD = 0.97, *p* = 0.03), and a 2025 systematic review of BFR in knee arthroplasty found significant improvements in muscle strength and functional outcomes in two of four RCTs that applied BFR preoperatively [33].

However, BFR is not appropriate in the 0–2 week postoperative window. A 2025 systematic review and meta-analysis of 11 RCTs specifically examining BFR in the early postoperative period (≤3 weeks post-ACLR) found no significant improvements in quadriceps cross-sectional area (SMD not significant) or maximal voluntary isometric contraction compared to control conditions [34]. A comparative meta-analysis of BFR versus standard rehabilitation after ACLR similarly found no advantage during the acute phase [35]. The mechanism of BFR requires sustained rhythmic contractions across multiple sets to accumulate sufficient metabolic stress and anabolic signaling, conditions that cannot be safely engaged when acute AMI prevents adequate voluntary activation and surgical wounds remain in the hemostatic phase. There is also a theoretical concern regarding elevated venous pressure on healing tissue in the immediate postoperative period, though serious adverse events in knee surgery populations appear to be rare [32]. BFR represents a potentially valuable mid-phase tool (typically from week 4–6 onwards) but does not address the immediate postoperative activation gap, and its use before adequate wound healing and volitional activation capacity is established cannot be recommended based on current evidence.

### 6.4. Electromyographic Biofeedback

EMG biofeedback provides real-time visual or auditory information about muscle activation amplitude, enabling patients to consciously modulate neuromuscular output in response to objective performance data. Three systematic reviews with meta-analyses provide converging quantitative evidence: Ananiás and colleagues (2024, 5 RCTs) found significant improvements in knee extension (SMD = −1.3, 95% CI −1.74 to −0.86) and quadriceps strength benefits in the majority of studies [36]; Xie and colleagues (2021, 6 RCTs) found significant ROM improvement (SMD = −0.48, 95% CI −0.82 to −0.14, *p* = 0.006, I^2^ = 37%), with no significant benefit for pain or physical function scores [37]; and Niyonkuru and colleagues (2025, 8 RCTs) identified EMG biofeedback as superior to home exercises, standard rehabilitation, and NMES for quadriceps strength and activation [38]. A systematic review published in Knee Surgery, Sports Traumatology, Arthroscopy confirmed superior quadriceps strength and functional outcomes in TKA patients, with the greatest benefit observed in the early postoperative period [39].

The therapeutic mechanism of EMG biofeedback aligns precisely with the neurophysiological targets of early rehabilitation: real-time activation feedback attenuates fear-of-movement responses, reinforces successful motor engrams, and sustains patient engagement during a phase characterized by high pain and psychological vulnerability [21]. It is the only existing modality with directly measured objective monitoring of activation quality. However, important limitations constrain its applicability: the requirement for specialized instrumentation, surface electrode arrays, signal amplifiers, and display interfaces, makes it non-portable and unsuitable for home deployment. There is also heterogeneity in EMG biofeedback protocols across studies, including variation in feedback modality (visual vs. auditory), threshold settings, and session frequency, which contributes to inconsistent findings across the literature. Biofeedback-assisted training is therefore currently limited to supervised clinical sessions, precisely the setting where patients are most likely to exercise adequately regardless of device support. The clinical challenge is translating the biofeedback principle into a portable, home-suitable format without loss of therapeutic efficacy.

### 6.5. Open and Closed Kinetic Chain Exercise

The historical concern that OKC knee extension generates anterior tibial shear forces harmful to the ACL graft has been substantially addressed by contemporary evidence. A 2024 systematic review and meta-analysis (9 RCTs) found that OKC exercises produced significantly superior quadriceps isokinetic strength compared to CKC exercises at three months (*p* = 0.03) and four months (*p* = 0.008), with early OKC producing greater pain reduction than late OKC (*p* < 0.003) without evidence of increased laxity [40]. A 2025 meta-analysis confirmed that OKC initiated at four weeks or later, following a CKC foundation in the first two weeks, produces beneficial effects on strength, function, and return-to-sport rates [41]. The 2023 Aspetar guideline now formally endorses OKC inclusion from four weeks onwards [6], resolving a longstanding clinical debate.

For the 0–2 week window, CKC exercises remain the evidence-supported foundation. The principal limitation shared with isometric exercise is the absence of any mechanism for objectively monitoring repetition quality, effort consistency, or progressive resistance calibration across home sessions. Both CKC and OKC protocols presuppose that the patient is exercising at a sufficient therapeutic intensity, an assumption that is difficult to verify in home settings. Despite their advantages in terms of accessibility and safety, the absence of objective monitoring represents the same fundamental gap identified for isometric exercise, and no RCT has specifically examined CKC-based home exercise adherence using objective tracking methods in the 0–2 week postoperative period.

### 6.6. Cryotherapy

Cryotherapy reduces tissue temperature to attenuate pain and swelling. A 2024 systematic review and meta-analysis of 31 RCTs demonstrated statistically significant pain reductions on postoperative days 1–3 after TKA (mean differences −0.59 to −0.86 on visual analogue scale), reduced opioid consumption, and improved early range of motion [42]. Beyond pain management, cryotherapy has a mechanistically distinct and important role in early rehabilitation: by reducing intra-articular pressure through vasoconstriction and attenuating inflammatory cytokine activity, cryotherapy reduces the afferent inhibitory drive responsible for AMI [3], creating a window of transiently reduced inhibition. An early-phase crossover RCT (n = 22 patients ≤ 10 days post-surgery) found that ice bag application produced a 38% increase in vastus medialis EMG activity during quadriceps setting and a 30% increase in maximum isometric knee extension strength compared to sham treatment [43], directly demonstrating the neuromuscular facilitation effect of cryotherapy in the early postoperative period.

These findings support cryotherapy as a synergistic adjunct rather than a primary rehabilitation intervention. Applied 15–20 min before exercise sessions, cryotherapy may enhance the effectiveness of subsequently performed volitional exercises by temporarily attenuating the AMI it does not itself resolve. However, the evidence for long-term functional benefit from cryotherapy alone is limited, and its primary value lies in creating the physiological conditions for more effective active rehabilitation, rather than directly producing motor re-learning. Continuous cold-flow devices have not been shown to produce superior outcomes compared to standard ice packs, supporting the use of less costly approaches in clinical practice [42].

### 6.7. Continuous Passive Motion

CPM was historically a standard of care following TKA, based on foundational experimental work demonstrating benefits of early joint movement on articular cartilage healing. Contemporary evidence does not support its routine use. An RCT of 80 TKA participants found no significant difference in objective range of motion or Knee Society Score between CPM plus active exercise and active exercise alone at any follow-up timepoint [44]. A subsequent RCT found that low-load active resistance training produced superior knee flexion range of motion and functional outcomes compared to passive CPM [45]. Systematic reviews of CPM in arthroplasty rehabilitation have similarly concluded that the evidence for routine CPM is insufficient to justify its routine cost and resource requirements.

CPM carries three fundamental limitations for the 0–2 week window: it is entirely passive, contributing nothing to voluntary motor re-learning or the reversal of AMI; it provides no objective performance data; and it is bulky and unsuitable for home deployment without professional assistance. Its continued use in some centers likely reflects institutional habit rather than evidence. The contemporary evidence base supports active exercise as the preferred approach, with CPM potentially reserved for specific clinical contexts such as patients with severe extension deficits or arthrofibrosis risk, rather than as a universal standard of care.

## 7. Synthesis of Current Interventions

Table 1 summarizes the evidence-based characteristics of each intervention across six clinically relevant dimensions: safety in the 0–2 week postoperative window, home-based usability, capacity to overcome AMI, requirement for volitional effort, objective monitoring capability, and progressive resistance. An additional column provides the approximate level of evidence (LoE) for each modality, based on the highest-quality study design available [25,27,28,33,35,37,43]. A consistent pattern is evident: no single existing modality simultaneously satisfies all six dimensions (Table 1).

Table 2 provides a structured summary of the key studies included in this review. Across all intervention categories, several cross-cutting observations merit comment. First, NMES and EMG biofeedback are the only modalities with direct objective monitoring of neuromuscular function, yet both require clinical infrastructure that limits home deployment. Second, isometric and kinetic chain exercises are the most accessible and widely used tools, yet lack the verification mechanisms that would allow clinicians to confirm therapeutic dosing in home settings. Third, BFR, while promising for mid-phase use, consistently fails to show benefit in the acute phase across multiple high-quality trials. Fourth, cryotherapy appears most valuable as an enabler of other modalities rather than as a direct rehabilitation intervention. Fifth, the evidence base is heterogeneous across interventions, with variability in protocol parameters, outcome measures, and follow-up durations contributing to inconsistency across studies, a limitation that should temper definitive conclusions about the relative effectiveness of individual modalities.

## 8. Discussion

This narrative review has synthesized evidence for seven early-phase knee rehabilitation interventions across all forms of knee surgery, with additional consideration of preoperative patient factors that modulate rehabilitation response. A clear evidence hierarchy emerges: NMES is supported by the highest-quality evidence (SMD 0.53–0.81) [25,26,27] but is fundamentally passive and infrastructure-dependent; EMG biofeedback is the only modality combining volitional activation with objective real-time monitoring yet cannot be deployed outside supervised settings [36,37,38,39]; isometric exercises are universally accessible but unmonitored; BFR shows no significant benefit in the acute phase [33,34]; and cryotherapy and CPM serve supporting rather than primary activation roles [42,43,44,45]. The overarching limitation clusters around two themes: the inability to deploy high-quality monitoring outside supervised settings, and the inability to combine volitional engagement with objective performance feedback in a portable format.

The significance of this collective gap extends beyond technical inconvenience. The early postoperative window may be a critical period for AMI intervention: AMI occurred in 36% of arthroplasty patients at two weeks in a prospective multicenter study [20], and cortical reorganization associated with sustained AMI can persist for more than two years if not targeted [21]. Quadriceps strength deficits established in this period contribute to a post-traumatic osteoarthritis rate estimated at approximately 5% within five years of ACLR [46] and up to 50% within two decades [4,5]. Current rehabilitation tools use this window incompletely, and the resulting persistent deficits, despite well-established guidelines, suggest that protocol adherence monitoring and home deployment capability are the critical bottlenecks rather than a lack of clinical knowledge.

The gap identified in this review points toward a specific design brief for future rehabilitation tools: portable, home-deployable, mechanically progressive, and incorporating an objective mechanism for verifying that each repetition meets a therapeutic activation threshold. Multiple technological pathways could address this brief, including pressure-based resistance mechanisms, wearable EMG systems, smart resistance bands with real-time force feedback, digitally monitored exercise platforms, telerehabilitation systems with therapist-supervised remote sessions, and pressure-sensitive mat systems. Each approach has distinct advantages and limitations: wearable EMG retains the direct neuromuscular measurement principle of biofeedback but raises questions of accuracy and calibration outside laboratory settings; digital platforms offer scalable remote monitoring but require patient access to technology and internet connectivity; pressure-based mechanical devices offer simplicity and robustness but rely on an indirect proxy for activation quality. Comparative evaluation of these approaches through well-designed RCTs, using quadriceps voluntary activation and strength as co-primary outcomes, should be a research priority. In this context, the author discloses that a patented rehabilitation device concept, the Knee Extensors Activation (KEA) Device (Australian Patent AU2024278483B1) [47], has been developed as one candidate approach incorporating adjustable resistance, real-time pressure monitoring, and objective repetition verification. This device has not been manufactured or clinically tested; it is mentioned for transparency only and its inclusion in this disclosure does not imply superior clinical effectiveness relative to the alternative approaches described above.

### 8.1. Implications for Clinical Practice

Until novel validated tools become available, clinicians should maximize the use of existing evidence-based strategies in combination, guided by individual patient risk profiles as described in Section 3. Cryotherapy applied 15–20 min before exercise sessions may reduce AMI severity and enhance the effectiveness of subsequently performed volitional exercises [43]. NMES should be prioritized in patients with severe AMI (Grade 2–3) who cannot generate volitional contractions, and should be initiated as early as postoperative day two where available [22,25]. EMG biofeedback, where clinical instrumentation is available, should be used to establish initial motor patterns before transitioning to home exercise protocols [36]. Adherence monitoring through regular telehealth check-ins and patient exercise logs partially mitigates the monitoring gap of unmonitored home exercise, though these strategies do not provide the per-repetition objective verification that would be ideal. For patients with identified preoperative risk factors, closer follow-up and more frequent supervised sessions in the first two weeks are warranted.

### 8.2. Future Research Priorities

Future research should prioritize three areas. First, well-powered RCTs evaluating novel devices or technology-assisted approaches that simultaneously address portability, volitional engagement, and objective monitoring should be designed with quadriceps voluntary activation and strength as co-primary outcomes, and should include longer-term follow-up to assess whether early activation improvements translate into reduced OA rates and reinjury risk. Second, implementation science research examining the specific barriers to home exercise adherence in the acute postoperative period would better define the device characteristics required for effective translation from clinic to home. Third, prospective studies examining the interaction between preoperative patient factors and early rehabilitation modality should be conducted to enable the development of evidence-based risk stratification tools, allowing clinicians to match intervention intensity and modality to individual patient need.

### 8.3. Limitations

This narrative review carries several important limitations that readers should consider. Conducted without a registered protocol, it is subject to selection and reporting bias; the absence of a formal systematic search and PRISMA flow diagram means that the evidence presented represents the authors’ synthesis rather than an exhaustive literature census. No formal quality appraisal tool was applied to included studies, and the level of evidence ratings in Table 1 are approximate rather than calculated through a standardized grading system. The review covers a broad range of interventions at necessarily summary depth; each modality’s literature is more extensive and nuanced than can be fully represented here. The 0–2 week timeframe focus means that evidence from later rehabilitation phases was selectively included. The review primarily addresses quadriceps activation; contributions of hamstring co-activation, hip abductor strength, and core neuromuscular control to early recovery are equally important clinically but were outside the defined scope. Finally, the author holds a patent for a device concept in the field reviewed; while this review was structured as independent evidence synthesis and the device is mentioned only briefly and with explicit transparency, readers should weigh the findings with this relationship in mind.

## 9. Conclusions

The first two postoperative weeks following knee surgery represent a period of notable neurological vulnerability and rehabilitation opportunity. AMI, initiated within hours of surgery across all knee surgery types, drives deficits that, if not actively counteracted, may propagate into persistent quadriceps weakness and premature joint degeneration. Preoperative patient-related factors including baseline quadriceps strength, age, BMI, and physical fitness modulate the severity of AMI and the capacity to respond to rehabilitation, and should be considered in clinical planning.

Current rehabilitation tools each address part of the challenge: NMES most effectively overcomes AMI but is passive and clinically dependent; isometric exercises are universally accessible but unmonitored; EMG biofeedback provides the closest approximation to an ideal tool but requires non-portable instrumentation; BFR is contraindicated in the acute phase; CPM provides limited benefit over active exercise. No existing modality simultaneously provides portability, volitional engagement, objective performance monitoring, and progressive resistance for home use from postoperative day one. This collective gap represents a clear, specific, and clinically important unmet need. Future randomized controlled trials evaluating novel integrated rehabilitation tools, including pressure-based devices, wearable EMG systems, and digitally monitored exercise platforms, are needed to determine whether addressing this gap produces measurable reductions in post-traumatic osteoarthritis and reinjury risk.

## 10. Patents

The author holds Australian Patent AU2024278483B1 for the Knee Extensors Activation (KEA) Device. The device has not been manufactured or subjected to clinical trials. Its mention in this manuscript is made for transparency only.

## Figures and Tables

**Table 1:** Characteristics of early-phase knee rehabilitation interventions (0–2 weeks postoperative) across six clinically relevant dimensions.

Intervention	Safe 0–2 Wks	Home-Based	Overcomes AMI	Volitional	Objective Monitoring	Progressive Resistance	Level of Evidence
Neuromuscular electrical stimulation	Yes	Partial a	Yes (passive)	No	No	Partial	I–II
Isometric exercise (quad set/SLR)	Yes	Yes	Partial	Yes	No	No	I–II
Blood flow restriction training	No	No	Partial	Yes	No	Yes	I–II
Electromyographic biofeedback	Yes	No	Yes	Yes	Yes	No	I–II
Closed/open kinetic chain exercise	Partial b	Yes	Partial	Yes	No	Partial	I–II
Cryotherapy	Yes	Yes	Partial c	No	No	No	I–II
Continuous passive motion	Yes	Partial d	No	No	No	Partial	I–II

SLR = straight leg raises; AMI = arthrogenic muscle inhibition; LoE = Level of Evidence (I = systematic review/meta-analysis of RCTs; II = RCT). Criteria definitions: Safe 0–2 Wks: no evidence of harm or contraindication in the immediate postoperative period based on current trial data. Home-Based: deployable without professional supervision in a home setting. Overcomes AMI: mechanism directly targets or bypasses the inhibited voluntary activation pathway. Volitional: requires active patient-generated muscle contraction. Objective Monitoring: provides real-time data on effort or activation quality during exercise. Progressive Resistance: allows systematic increase in mechanical challenge across rehabilitation sessions. Classification criteria for “Partial”: a = Home NMES units exist but require supervised calibration; passive mechanism does not provide performance feedback; b = CKC is safe from week 0 but OKC carries theoretical risk before week 4; c = Cryotherapy addresses AMI indirectly through effusion reduction, not via direct neuromuscular activation; d = CPM requires equipment delivery and setup assistance. Yes = criterion fully met by current evidence; No = criterion not met; Partial = criterion met only under specific conditions as defined above.

**Table 2:** Summary of key studies included in this review, by intervention category.

Author, Year	Design	Population	Intervention	Outcomes	Key Findings
Rice & McNair, 2010 [3]	Literature review	Knee joint pathology	AMI mechanisms	Quadriceps activation	20–30 mL effusion reduces quadriceps activation by up to 50% via presynaptic inhibition and corticospinal pathways
Sonnery-Cottet et al., 2022 [2]	Prospective cohort	Knee ligament surgery (n = 200+)	AMI classification	VMO contraction, ROM	Proposed Grades 0–3 classification; Grade 2–3 associated with flexion contracture and poor early outcomes
Le Guen et al., 2025 [20]	Prospective multicenter	TKA (n = 316)	AMI assessment	AMI prevalence	36% AMI ≥ 1 at 2 weeks; female gender (OR = 2.81), pain (OR = 13.57), and positional habits were independent predictors
Lepley & Lepley, 2022 [21]	Narrative review	ACL injury/surgery	AMI mechanisms	Corticospinal excitability	Intracortical inhibition and corticospinal hyperexcitability persist >2 years without targeted early intervention
Stevens-Lapsley et al., 2012 [22]	RCT (n = 66)	TKA	NMES 48h post-op vs. control	Quad strength, function	NMES significantly attenuated quadriceps loss; improved stair-climb, TUG, 6MWT at 3.5 weeks; effects to 1 year
Li Z et al., 2025 [25]	Systematic review + meta-analysis (11 RCTs, n = 402)	ACL surgery	NMES + standard vs. standard	Quadriceps strength, Lysholm	NMES significantly improved strength (SMD ≤ 0.53–0.59; *p* < 0.001); earlier initiation (≤1 week) produced greater benefit
Peng L et al., 2021 [27]	Systematic review + meta-analysis (14 RCTs)	TKA	NMES vs. standard	Quad strength, pain, function	NMES improved strength at <1 month (SMD 0.81), 1–2 months (SMD 0.55), 12–13 months (SMD 0.46)
Sakai et al., 2025 [28]	RCT (n = 37, 60 knees)	TKA	NMES from POD2 vs. control	Isometric strength, ROM, TUG	NMES group showed significantly better quadriceps strength at 4 and 8 weeks postoperatively
Labanca L et al., 2022 [26]	Systematic review (14 studies)	TKA	Quadriceps NMES	Quad strength	All studies reported higher quadriceps strength with NMES particularly early after TKA
Shaw et al., 2005 [29]	RCT (n = 103)	ACLR	SLR + isometric contractions weeks 0–2 vs. no exercise	ROM, laxity, Cincinnati scores	Exercise group: significantly better ROM (*p* = 0.01–0.04), lower laxity at 6 months, better functional scores
Solie et al., 2024 [30]	Clinical commentary + framework	ACLR	Evidence-based quadriceps exercise framework	Quad size, strength	Confirmed quad sets and SLR as cornerstones of immediate rehabilitation; favorable EMG patterns with minimal ACL strain
Bakaa N et al., 2022 [31]	Cross-sectional (n = 204)	TKA	Adherence survey	Exercise completion rates	Completion rates as low as 30%; day-to-day pain, mood, and self-efficacy are primary adherence drivers
Hughes L et al., 2019 [32]	Systematic review + meta-analysis	Musculoskeletal rehabilitation	BFR training	Strength, hypertrophy	BFR at 20–40% 1RM produces comparable gains to high-load training at 70–80% 1RM
Li X et al., 2025 [34]	Systematic review + meta-analysis (11 RCTs)	ACLR	BFR vs. standard ≤ 3 weeks	ACSA, MVIC, IKDC	No significant early (<3 weeks) benefit on quadriceps CSA or MVIC; possible mid-term IKDC benefit
Chen et al., 2025 [33]	Systematic review + meta-analysis	Middle-aged/elderly knee conditions	BFR low-load training	Muscle strength, pain	BFR significantly enhanced postoperative muscle strength (SMD = 0.97, *p* = 0.03) in preoperative cohorts
Ananiás et al., 2024 [36]	Systematic review + meta-analysis	ACLR	EMG biofeedback vs. standard	Knee extension, quad strength	Significant improvement in knee extension (SMD = −1.3, 95% CI −1.74 to −0.86); quadriceps benefits in majority of studies
Xie et al., 2021 [37]	Systematic review + meta-analysis (6 RCTs)	Knee surgery	EMG biofeedback vs. other rehab	ROM, pain, function	EMG biofeedback significantly improved ROM (SMD = −0.48, *p* = 0.006); no significant pain or function benefit
Karaborklu S et al., 2022 [38]	Systematic review (8 RCTs)	Orthopaedic knee surgery	EMG biofeedback vs. standard/NMES	Strength, activation	EMG biofeedback superior to home exercises, standard rehab, and NMES for quadriceps strength and activation
Pfeufer et al., 2019 [39]	Systematic review (11 studies)	TKA	Biofeedback training vs. usual care	Quad strength, function	Biofeedback training produced superior quadriceps strength and functional outcomes; benefits greatest early post-op
Pamboris & Mohagheghi, 2024 [40]	Systematic review + meta-analysis (9 RCTs)	ACLR	OKC vs. CKC	Strength, pain, laxity	OKC produced superior isokinetic strength at 3 months (*p* = 0.03) and 4 months (*p* = 0.008); no laxity difference
Fontanier V et al., 2025 [41]	Systematic review + meta-analysis	ACLR	OKC weeks 0–2 vs. later	Strength, function, RTS	OKC from ≥4 weeks after CKC foundation produces beneficial effects; confirms CKC dominant in 0–2 week window
Liang et al., 2024 [42]	Systematic review + meta-analysis (31 RCTs)	TKA	Cryotherapy vs. control	VAS pain, opioid use, ROM	Significant pain reduction days 1–3 (MD −0.59 to −0.86 VAS); reduced opioids; no advantage for continuous vs. standard cryo
Loro et al., 2019 [43]	Crossover RCT (n = 22)	Early post-surgical knee	Ice bag 20 min vs. sham	VM EMG, isometric strength	Cryotherapy produced 38% increase in VM EMG activity and 30% increase in maximal isometric strength vs. sham
Richter et al., 2021 [44]	RCT (n = 80)	TKA	CPM + active exercise vs. active exercise	ROM, KSS	No significant difference in ROM or Knee Society Score between groups
Jacksteit R et al., 2021 [45]	RCT	TKA	Low-load resistance vs. passive CPM	ROM, function	Active low-load training produced superior knee flexion ROM and functional outcomes vs. CPM
Ueda Y et al., 2023 [13]	Prospective cohort (n = 646)	ACLR	Predictors of quadriceps recovery	Quad LSI at 1 year	Age > 22.5y, female sex, BMI > 24.3, lower sport level, and preoperative QI < 63.3% independently predicted poor quadriceps recovery
Kitaguchi T et al., 2020 [12]	Case–control (n = 221)	ACLR (competitive athletes)	Preoperative QI vs. RTS	RTS at 1 year	Preoperative QI < 66% predicted unsuccessful RTS (OR = 1.68 per 10-unit increase; AUC = 0.74)
Filbay & Grindem, 2019 [4]	Narrative review	ACL injury	Evidence-based recommendations	OA, reinjury, function	Quadriceps strength at 12 weeks explains ~47% variance in return-to-sport strength; persistent weakness linked to OA
Øiestad BE et al., 2019 [5]	Prospective cohort	Adults with prior knee injury	Quadriceps weakness	Cartilage thickness	Quadriceps weakness associated with accelerated cartilage thinning in medial tibiofemoral compartment over 24 months

ACLR = ACL reconstruction; TKA = total knee arthroplasty; RCT = randomized controlled trial; SMD = standardized mean difference; QI = quadriceps index; LSI = limb symmetry index; VMO = vastus medialis oblique; ROM = range of motion; KSS = Knee Society Score; TUG = timed up and go; RTS = return to sport; OA = osteoarthritis; MVIC = maximal voluntary isometric contraction; ACSA = anatomical cross-sectional area; IKDC = International Knee Documentation Committee; POD = postoperative day; 6MWT = 6 min walk test.

## Data Availability

No new data were created or analyzed in this study. Data sharing is not applicable to this article.

## Abbreviations

The following abbreviations are used in this manuscript:

ACL	Anterior Cruciate Ligament
AMI	Arthrogenic Muscle Inhibition
BFR	Blood Flow Restriction
CKC	Closed Kinetic Chain
CPM	Continuous Passive Motion
EMG	Electromyographic
GRADE	Grading of Recommendations, Assessment, Development and Evaluations
KEA	Knee Extensors Activation
LoE	Level of Evidence
NMES	Neuromuscular Electrical Stimulation
OKC	Open Kinetic Chain
QI	Quadriceps Index
RTS	Return to Sport
SLR	Straight Leg Raise
SMD	Standardized Mean Difference
TKA	Total Knee Arthroplasty
VMO	Vastus Medialis Oblique
VL	Vastus Lateralis
